# Protocols for the delivery of small molecules to the two-spotted spider mite, *Tetranychus urticae*

**DOI:** 10.1371/journal.pone.0180658

**Published:** 2017-07-07

**Authors:** Takeshi Suzuki, María Urizarna España, Maria Andreia Nunes, Vladimir Zhurov, Wannes Dermauw, Masahiro Osakabe, Thomas Van Leeuwen, Miodrag Grbic, Vojislava Grbic

**Affiliations:** 1 Department of Biology, The University of Western Ontario, London, Ontario, Canada; 2 Laboratory of Agrozoology, Department of Crop Protection, Faculty of Bioscience Engineering, Ghent University, Gent, Belgium; 3 Laboratory of Ecological Information, Graduate School of Agriculture, Kyoto University, Kyoto, Japan; 4 Department of Evolutionary Biology, Institute for Biodiversity and Ecosystem Dynamics, University of Amsterdam, Amsterdam, the Netherlands; 5 Universidad de La Rioja, Logroño, Spain; Ecole Normale Superieure, FRANCE

## Abstract

The two-spotted spider mite, *Tetranychus urticae*, is a chelicerate herbivore with an extremely wide host range and an extraordinary ability to develop pesticide resistance. Due to its responsiveness to natural and synthetic xenobiotics, the spider mite is becoming a prime pest herbivore model for studies of the evolution of host range, plant-herbivore interactions and mechanisms of xenobiotic resistance. The spider mite genome has been sequenced and its transcriptional responses to developmental and various biotic and abiotic cues have been documented. However, to identify biological and evolutionary roles of *T*. *urticae* genes and proteins, it is necessary to develop methods for the efficient manipulation of mite gene function or protein activity. Here, we describe protocols developed for the delivery of small molecules into spider mites. Starting with mite maintenance and the preparation of the experimental mite populations of developmentally synchronized larvae and adults, we describe 3 methods for delivery of small molecules including artificial diet, leaf coating, and soaking. The presented results define critical steps in these methods and demonstrate that they can successfully deliver tracer dyes into mites. Described protocols provide guidelines for high-throughput setups for delivery of experimental compounds that could be used in reverse genetics platforms to modulate gene expression or protein activity, or for screens focused on discovery of new molecules for mite control. In addition, described protocols could be adapted for other Tetranychidae and related species of economic importance such as *Varroa*, dust and poultry mites.

## Introduction

The two-spotted spider mite (TSSM) *Tetranychus urticae* Koch (Acari: Tetranychidae) is an important agricultural pest worldwide. It is a chelicerate herbivore that feeds on an extremely wide host range, including over 100 agricultural crops [1]. Furthermore, *T*. *urticae* populations have the highest occurrence of pesticide resistance among arthropods in agricultural habitats [2]. *T*. *urticae* genome was recently sequenced [3] and several genomic databases of transcriptome profiles characterizing *T*. *urticae* developmental stages and its responsiveness to a variety of xenobiotic and abiotic stresses are available at ORCAE (http://bioinformatics.psb.ugent.be/orcae/) [4]. In addition, several reports describe both forward and reverse genetics approaches to identify genes of interest [5–7].

The rapid responsiveness to natural and synthetic xenobiotics makes *T*. *urticae* a prime model for studies of the evolution of host range, plant-herbivore interactions and mechanisms of xenobiotic resistance. However, to fully exploit the potential of spider mite as a model experimental system, it is necessary to develop methods for the efficient manipulation of mite gene function. This includes the delivery of different types of molecules that modulate gene expression, such as double stranded RNAs (dsRNA), morpholinos, or transgenes, or molecules that alter protein activity, including natural or synthetic chemicals that act as agonists or antagonists. Such methods must be robust, reproducible, cost-effective, and designed to record the effects of small molecules on otherwise physiologically normal mites. In addition, these bioassays should be applicable to a large population of mites, so that the effects of small molecules on mite physiology can be quantified phenotypically or using biochemical or molecular analyses.

Several approaches have been described for the delivery of small molecules to arthropods. RNA interference (RNAi) triggered by dsRNA has become an important reverse genetics and biotechnological tool for arthropod research and pest control [8,9]. Delivery of dsRNA through artificial diet and microinjections into the hemolymph have been the most widely used methods in insects [10–14]. Alternatively, soaking has been routinely applied to deliver dsRNA into nematodes [15–17].

Even though an artificial diet has been developed for *T*. *urticae* decades ago [18–20], it has not been used for the delivery of dsRNA. dsRNA microinjection and feeding on leaf discs floating on dsRNA solution have been used instead [6,7,21]. However, these methods are not suited for high-throughput applications: microinjection introduces a non-specific stress caused by mechanical damage and is cumbersome because adult female mites are ≤ 0.5 mm long; and the leaf-floating method requires large (>20 μg per individual sample) amounts of dsRNA. Other methods for the delivery of small molecules include spraying and leaf dip bioassays and have been most frequently used for the application of synthetic chemicals, e.g. pesticides [22], to a wide range of arthropods including *T*. *urticae*. But, these methods require considerable volume of the experimental solution and are not suitable for high-throughput setups.

We report 3 different methods for the delivery of small molecules to *T*. *urticae* including artificial diet, leaf coating, and soaking protocols. They are compatible with mites at all developmental stages and can be adapted for high-throughput screens. Small molecule delivery was validated with the demonstration of tracer dyes accumulation in the mite body upon application with the 3 protocols. The protocols described in detail here are a prerequisite for better understanding of mite biology and for the development of novel compounds aimed at spider mite pest control.

## Materials and methods

**Fig 1:**
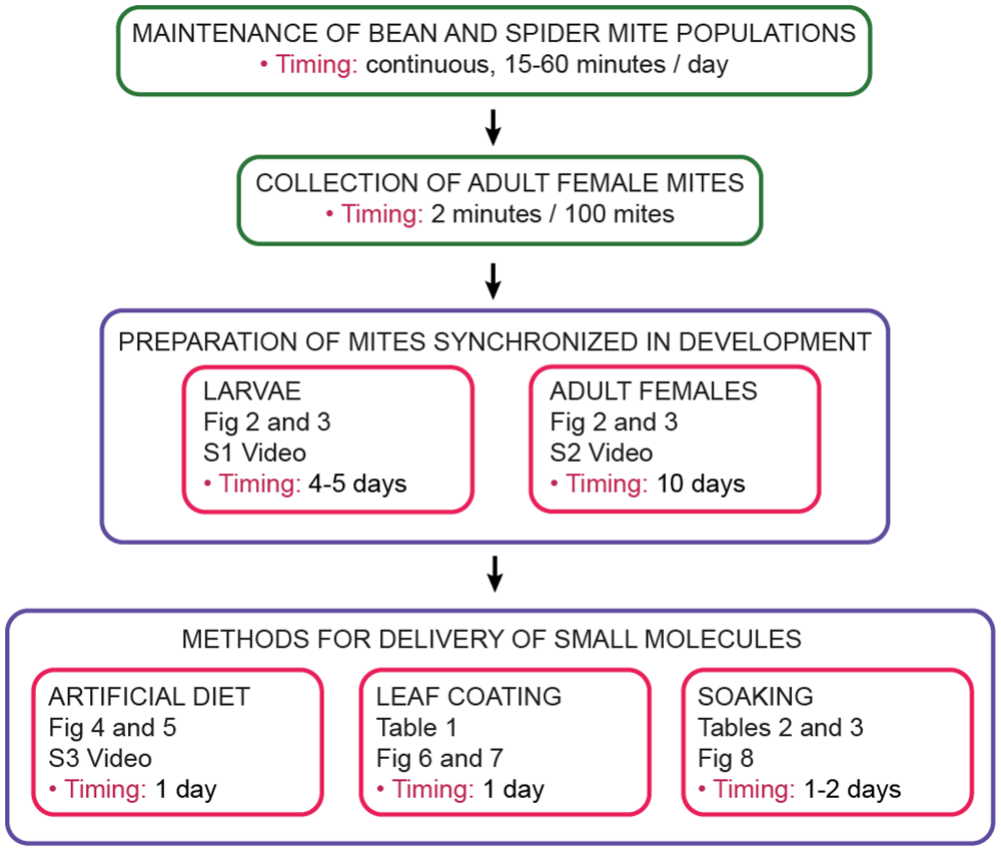
Flowchart of the procedure. The main steps of the procedure include maintenance of bean and spider mite populations (Step 1), collection of the adult female mites (Step 2), preparation of *T*. *urticae* larvae and adult female mites tightly synchronized in their development (Step 3) and methods for delivery of small molecules (Step 4). The respective figures, tables, supplementary figures and supplementary videos are referenced.

An overview of methods for the delivery of small molecules to *T*. *urticae* (artificial diet, leaf coating and soaking) is shown in Fig 1. The first critical step, irrespective of the delivery method, is the preparation of uniform mite populations. The procedure thus includes the rearing of spider mites on bean plants (Step 1) from which adult female mites are collected (Step 2). Adult female mites are used to initiate experimental populations of larvae and adult mites that are tightly synchronized in their development (Step 3). Larvae and adults are subsequently used in separate delivery protocols named: artificial diet, leaf coating and soaking (Step 4).

### 1. Maintenance of spider mite populations on bean nursery plants

(**Timing:** continuous, with weekly renewal of plant batches)

#### Materials and equipment

1. California red kidney beans (Phaseolus vulgaris L.) (Stokes, Thorold, ON)
2. Soil (PRO-MIX^®^ BX MYCORRHIZAE^™^; Premier Tech, Rivière-du-Loup, QC, or similar)
3. Suitable 3.5 inch (or similar) pots and trays
4. Stock population of T. urticae (London strain, Grbic lab)
5. Climate controlled chamber

The stock population of the two-spotted spider mite is maintained on California red kidney beans in a climate controlled chamber at 26°C, with a 50% relative humidity (RH), a photoperiod of 16h:8h light:dark (16:8 L:D) and a photosynthetic photon flux density of 150 μmol m^−2^ s^−1^ using Philips Fluorescent Plant Light bulbs (Philips, Amsterdam, Netherlands). The generation time of the London reference mite strain under these conditions is 8–9 days. This time may differ for other mite populations and other environments. In this environment, bean plants reach the 3-4-true-leaf stage suitable for the inoculation by mites in 2 to 3 weeks. The mite rearing is continuous and requires a fresh batch of bean plants once a week.

#### Stepwise procedure

1.1. Wet soil with tap water and lightly pack the water-soaked soil into pots.
1.2. Sow seeds (1–2 seeds per pot) and grow bean plants in the environment indicated above, until plants reach the 3–4-true-leaf stage. Water pots/plants every other day with tap water.
1.3. Intermix fresh plants (~15 pots in the 18 pot tray) with ~3 pots of the infested plants (for illustration see [23]). Mites will rapidly colonize fresh plants.
1.4. Remove old infested bean plants every 7–10 days.

#### Note

Mite population should be carefully monitored to never become too dense otherwise bean plants will be consumed too rapidly and mites will disperse at the risk of contaminating the rearing area.

### 2. Collection of adult female mites

(**Timing:** 1–2 minutes to collect 100 adult female mites)

#### Materials and equipment

1. Modified aquarium air pump with inverted air flow (Whisper 10–30; Tetra, Blacksburg, VA) or a vacuum line
2. 1 mL polypropylene pipette tip
3. Wipe (Kimwipe; Kimberly-Clark, Irving, TX)
4. 1.5 mL microcentrifuge tube
5. Flexible clear plastic tubing
6. Dissecting microscope

The vacuum system consisting of a 1 mL polypropylene tip, a piece of Kimwipe, a flexible clear plastic tubing and the air pump are shown in S1A Fig and have been previously described [23]. Briefly, attach a 1 mL pipette tip to the plastic tubing with a 1.5 mL microcentrifuge tube cut at the bottom as an adapter between the pump/vacuum tube and the tip. Place the piece of Kimwipe between the pipette tip and the tube to keep collected mites inside the tip.

#### Stepwise procedure

2.1 Intermix fresh bean plants with infested bean plants (ratio of ~2:1) in the afternoon and leave overnight (O/N). Adult mites will rapidly colonize fresh plants.
2.2. In the morning of the following day, collect the required number of mites directly from newly added bean leaves with the vacuum system using a dissecting microscope to visualize the mites (for illustration see [23]).
2.3. Remove the pipette tip from the tube, making sure that the piece of Kimwipe at the back of the pipette tip is undisturbed and transfer the collected mites into a new 1.5 mL microcentrifuge tube by tapping the tip.

#### Note

1. *T*. *urticae* displays sexual dimorphism: females are bigger than males and have rounded posterior end. Caution is also required to distinguish between the female deutonymphs (last nymph stage) that are smaller but otherwise morphologically similar to adult female mites. For best practice, identify the successive mite developmental stages and train yourself for the recognition of the adult female mites prior to mite collection.
2. Collection of adult mites is hampered by the presence of silk that mites spin on the leaf surface and mites entangled in silk may eventually die. To avoid silk contamination, recover adults from fresh bean plants that were intermixed with infested plants the day before collection. As adult mites are the most mobile developmental stage, the fresh bean plants will mainly carry adults and their leaves will not yet be densely covered with silk.

### 3. Preparation of mites synchronized in development

Submergence in water and high humidity were reported to inhibit spider mite egg hatching [24] and molting [25], respectively. We simplified these previously described methods while improving the synchronization of mite embryo and nymph development, with the aim to produce large cohorts of larvae and adults that are coordinated in their development. The procedures are detailed in the following 2 subsections, for larvae and adults, respectively.

#### Materials and equipment

1. Polystyrene cup (94 mm in diameter, 57 mm in depth)
2. Polyethylene lid with and without venting holes (referred to as vented and non-vented lids, respectively) (V-9, As-one, Osaka, Japan) (S1B and S1C Fig)
3. Gas-permeable filters (0.45 micron pore size; Milliseal; EMD Millipore, Billerica, MA)
4. Cotton wool
5. Forceps
6. Leaf puncher (Fujiwara Scientific, Tokyo, Japan)
7. 35-mm Petri dish
8. Tapered brush (Interlon 1026-3/0; Maruzen Artist Materials & Works, Tokyo, Japan)
9. Climate controlled chamber

To create vented-lids punch 4 holes (10 mm in diameter) in the lid with the leaf puncher and cover holes with gas-permeable filters (S1B and S1C Fig).

### 3.1. Production of developmentally synchronized larvae

(**Timing:** 4–5 days)

#### Stepwise procedure

**Fig 2:**
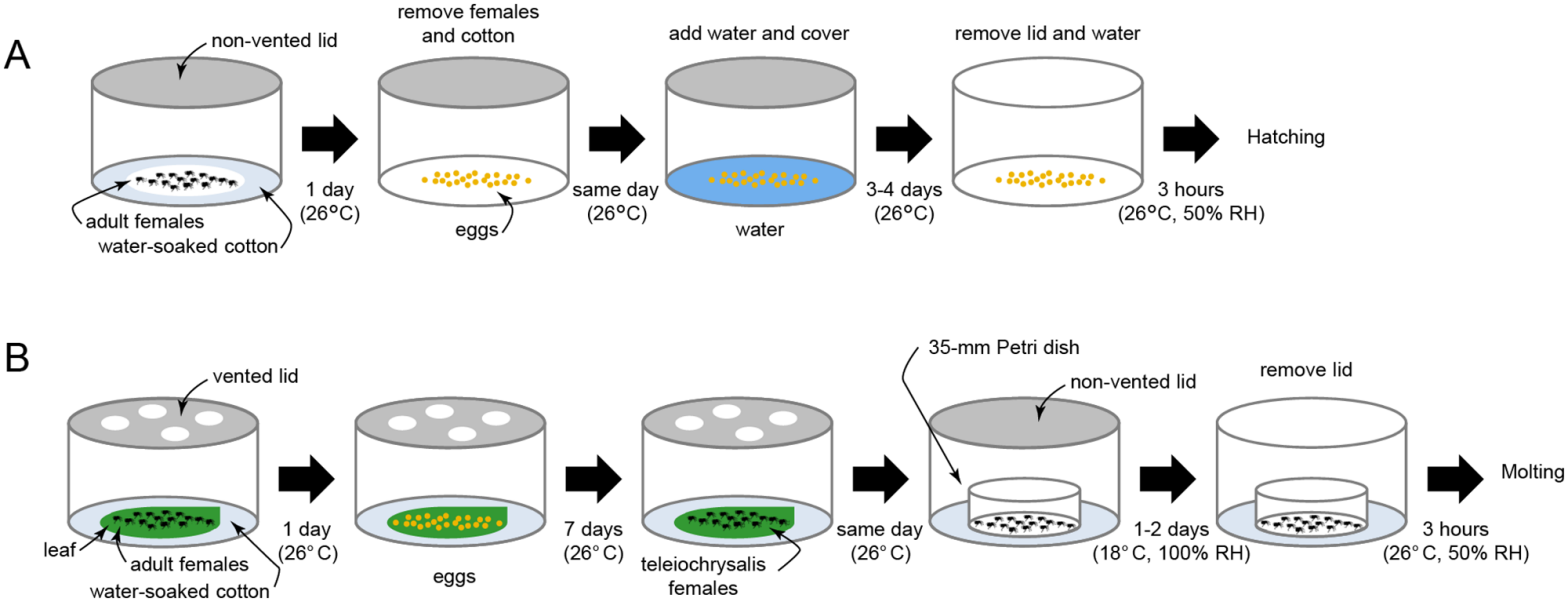
Preparation of mites synchronized in development. (A) Synchronization of larval emergence. Schematic overview of the protocol for the synchronous hatching of spider mite eggs. (B) Synchronization of adult emergence. Schematic overview of the protocol for the synchronous molting of spider mite deutonymphs, which is the last nymph stage in mite development.

This protocol is outlined in Fig 2A.

3.1.1. Create an area (approx. 50 mm in diameter) fenced with water-soaked cotton at the bottom of the cup.
3.1.2. Transfer ~500 adult females (collected as described in Section 2) into the area and cover the cup with a non-vented lid.
3.1.3. Incubate the adult females at 26°C for 1 day. During that time females will lay eggs (mites can lay eggs in the vapor saturated environment).
3.1.4. Remove the water-soaked cotton with forceps and all the adult females with vacuum suction (as described in Section 2).
3.1.5. Add water (15 mL) immediately to completely cover the 1-day-old eggs laid at the bottom of the cup.
3.1.6. Cover the cup with the non-vented lid and incubate at 26°C for 3 to 4 days.
3.1.7. After this period, remove water from the cup with a pipette.
3.1.8. Incubate the eggs in the same cup, but with no lid, at 26°C and 50% RH.

### 3.2. Production of developmentally synchronized adults

(**Timing:** 9–10 days)

#### Stepwise procedure

This protocol is outlined in Fig 2B.

3.2.1. Place a fresh detached bean leaf onto water-soaked cotton (80 mm in diameter) in the cup.
3.2.2. Transfer 50 adult female mites onto the leaf and cover the cup with the vented lid.
3.2.3. Incubate the adult females laying eggs on the leaf at 26°C for 1 day.
3.2.4. Remove adult females from the leaf with vacuum suction (as described in Section 2).
3.2.5. Cover the cup with the vented lid and incubate at 26°C for 7 days.
3.2.6. After 7 days, collect quiescent deutonymph (teleiochrysalis) females and carefully transfer them into a 35-mm Petri dish with a tapered brush without damaging mites.
3.2.7. Place the 35-mm Petri dish on water-soaked cotton in the cup.
3.2.8. Cover the cup with the non-vented lid to maintain teleiochrysalis in water-saturated air and incubate at 18°C for 1 to 2 days.
3.2.9. Remove the lid and place the cup at 26°C and 50% RH for 3 h.

### 4. Methods for delivery of small molecules

#### 4.1. Artificial diet

Two holidic diets (i.e. with chemically defined compositions) that were originally formulated for aphids [26,27] were tested for their ability to support mite development. The diet described by Prosser and Douglas [26] was tested in its original formulation. The diet formulated by Febvay *et al*. [27] was diluted 30-fold in water as described in Jonckheere *et al*. [28]. While these diets were able to support spider mite larvae and adults for a week, they did not support molting and larval transition to the nymph stage (data not shown).

We thus tested 2 additional meridic diets (i.e. including non-chemically defined constituents such as casein or wheat germs). The first, has been formulated by Gotoh *et al*. [29] for *Panonychus citri*, a mite species belonging to the same Tetranychidae family as *T*. *urticae*. However, this diet failed to support developmental transition of *T*. *urticae* larvae to protonymph, stressing that this developmental transition is a crucial step toward the establishment of an appropriate artificial diet. The second meridic diet we tested was formulated by Van der Geest *et al*. [18]. These authors reported that diet sustains the entire *T*. *urticae* life cycle, from larvae to adult. Indeed, we confirmed mite progression through all developmental stages while feeding on this diet and it is presented below.

#### Materials and equipment

**4.1.1. Reagents**
1. 0.1% Triton X-100 aqueous solution
2. 100 ng/μL Alexa Fluor 488 fluorescent dye (Thermo Fisher Scientific, Waltham, MA)
3. 50% glycerol in phosphate-buffered saline (PBS) solution (v/v) [30]
4. 3% blue food dye (erioglaucine; McCormick, Sparks Glencoe, MD)

Composition of chemicals required for the preparation of 100 mL of the artificial diet described in [18] is shown in S1 Table.

#### Preparation of a diet (100 mL)

4.1.1.1. Dissolve wheat germ powder and casein sodium salt in 50 mL of distilled water. Autoclave twice for 20 min at 120°C and reserve for later. **Note**: It has been proposed that autoclaving changes certain physical or chemical properties of component(s) in wheat germ powder and/or casein sodium salt that are required for successful mite progression through the life cycle [18]. Thus, this step should not be modified.
4.1.1.2. Weigh amino acids and dissolve them in 30 mL of warm (around 40°C) distilled water. Let solution cool to room temperature (RT).
4.1.1.3. Once the amino acids solution is at RT, add vitamins, mineral salts, glucose and cholesterol and adjust the pH to 6–6.5 with KOH.
4.1.1.5. Combine the amino acids, the wheat germ and casein solutions, and adjust volume to 100 mL with double distilled water. Filter sterilize with 0.2 μm filter.
4.1.1.6. Aliquot the artificial diet in 2 mL batches in sterile microcentrifuge tubes and preserve at 4°C for up to 3 months.

**4.1.2. Equipment**
1. A custom built vacuum device (S1D Fig), consisting of a 96-hole well plate (plate thickness 4.2 mm, hole diameter 4.5 mm) fitted on a vacuum manifold plate (Analytical Research Systems, Florida, USA)
2. Welch DryFast 2014B-1 (Gardner Denver Wayne, PA) or similar vacuum pump
3. Cabinet with the controlled environment
4. Parafilm^®^ M
5. Scotch tape
6. Source of UV light such as laminar flow hood sterilization light
7. 24-well culture plates
8. Climate controlled chamber

#### Stepwise procedure

4.1.2.1. Irradiate a piece of Parafilm^**®**^ M (10x20 cm) with UV light for 20 min to achieve at least 30,000 μJ/cm^**2**^ irradiation dose [31].
4.1.2.2. Place the Parafilm^®^ M over a custom built vacuum device pre-cleaned with 70% ethanol and turn on the vacuum pump reaching 20 psi to create Parafilm^®^ M hemispheres. Use sterile pestles if needed to aid the hemisphere formation.
4.1.2.3. Fill each hemisphere with 45 μL of the diet. An aliquot of the solution of small molecules or tracer dyes can be supplemented to the artificial diet.
4.1.2.4. Seal hemispheres with Scotch tape.
4.1.2.5. Remove the 96—Parafilm^®^ M hemispheres from the plate and cut individual hemispheres.
4.1.2.6. Fill wells in the 24-well culture plate with 1 mL of water to create a water barrier and prevent mite escapes.
4.1.2.7. Float individual hemispheres in each well, S1E Fig.
4.1.2.8. Place 1 adult or 5 larvae (as prepared with Protocol 3) per hemisphere and incubate them at 26°C, 50% RH, and 16:8 L:D photoperiod.

### 4.2. Leaf coating method

#### Materials and equipment

1. Polystyrene cup (94 mm in diameter, 57mm in depth)
2. Polyethylene lid with venting holes (vented lids) (V-9; As-one, Osaka, Japan) (S1C Fig)
3. Silwet L-77 (Lehle Seeds, Cat No VIS-01)
4. 0.1% Triton X-100 aqueous solution
5. 100 ng/μL Alexa Fluor 488 fluorescent dye (Thermo Fisher Scientific, Waltham, MA)
6. 50% glycerol in phosphate-buffered saline (PBS) solution (v/v) [30]
7. 3% blue food dye (erioglaucine; McCormick, Sparks Glencoe, MD)
8. Bean plants/leaves
9. Filter paper
10. Leaf puncher (Fujiwara Scientific, Tokyo, Japan)
11. Forceps
12. Tapered brush (Interlon 1026-3/0; Maruzen Artist Materials & Works, Tokyo, Japan)
13. Climate controlled chamber

#### Stepwise procedure

4.2.1. Prepare an experimental solution adding 0.025% Silwet L-77 just before its application on bean leaf discs (10 mm in diameter, cut with the leaf puncher). Solution can be supplemented with small molecules or tracer dyes.
4.2.2. Add a drop of 6 μL of experimental solution to the leaf disc placed in a Petri dish.
4.2.3. Let solution spread on the leaf surface (use pipette tip to help the spread if needed); let leaf disc surface dry, but avoid wilting.
4.2.4. Transfer surface-dried leaf discs to cups with vented lid that contain filter paper placed on the water-soaked cotton.
4.2.5. Transfer synchronized larvae or adults onto the leaf discs by brush and incubate at 16:8 L:D, 26°C.
4.2.6. Replace leaf discs every other day.

### 4.3. Soaking

#### Materials and equipment

1. 100 ng/μL Alexa Fluor 488 fluorescent dye (Thermo Fisher Scientific, Waltham, MA)
2. 3% blue food dye (erioglaucine; McCormick, Sparks Glencoe, MD)
3. Tween 20
4. 50% glycerol in phosphate-buffered saline (PBS) solution (v/v) [30]
5. A 8-cap strip for 0.2-mL PCR 8-tube strips
6. Tape
7. 90-mm Petri dish
8. 1.5-mL microcentrifuge tube
9. Polystyrene cup (94 mm in diameter, 57 mm in depth)
10. Polyethylene lid with venting holes (S1F Fig)
11. Tapered brush (Interlon 1026-3/0; Maruzen Artist Materials & Works, Tokyo, Japan)
12. Micropipette
13. Bean plants/leaves
14. Leaf puncher (Fujiwara Scientific, Tokyo, Japan)
15. Filter paper
16. Cotton wool
17. Forceps
18. Climate controlled chamber

#### Stepwise procedure

**4.3.1. Soaking larvae**
4.3.1.1. Prepare an 8-cap strip for 0.2-mL PCR 8-tube strips and tape it onto a 90-mm Petri dish (S1F Fig), each cap being a soaking reservoir.
4.3.1.2. Add 25 μL of soaking solution containing 0.1% Tween 20 and compounds of interest and/or tracer dyes into each cap.
4.3.1.3. Place newly-hatched larvae onto surface of the soaking solution in the cap (25 larvae/cap) with the brush. If some larvae float on the surface of the soaking solution, sink them by tapping the Petri dish.
4.3.1.4. Cover Petri dish with its lid.
4.3.1.5. Incubate the immersed larvae in the soaking solution at 20°C for 4 h.
4.3.1.6. Slowly remove the soaking solution with a micropipette.
4.3.1.7. Draw the washing solution including the larvae with a micropipette and transfer larvae onto a detached bean leaf placed on water-soaked cotton.
4.3.1.8. Remove the excess of washing solution surrounding the larvae with the micropipette. Let larvae dry, recover and start moving.
4.3.1.9. Prepare bean leaf discs (10 mm in diameter) with a leaf puncher and place the discs on water-soaked cotton in the cup.
4.3.1.10. Transfer the recovered larvae from the detached leaf onto the leaf discs (≤ 5 larvae/disc) with the brush.
4.3.1.11. Cover the cup with the vented lid and incubate at 16:8 L:D and 26°C.

**4.3.2. Soaking adults**
4.3.2.1. Collect 10 to 50 newly-molted adults with the vacuum system (as described in Protocol 2) and place them in a 1.5-mL microcentrifuge tube.
4.3.2.2. Add 10 to 50 μL (1 μL/mite) of 0.1% Tween 20 or Triton X-100 soaking solution containing a compound of interest and/or tracer dyes in the tube. If some adults float on the surface of the solution, sink them by tapping the tube.
4.3.2.3. Incubate the immersed adults in the soaking solution at 20°C for 24 h.
4.3.2.4. Slowly remove the soaking solution.
4.3.2.5. Add 100 μL of 0.1% Tween 20 or Triton X-100 washing solution.
4.3.2.6. Draw the washing solution with the adult mites and transfer them onto a bean leaf with a micropipette.
4.3.2.7. Remove the excess fluid surrounding the adults with a micropipette. Let mites dry, recover, and start moving.
4.3.2.8. Prepare bean leaf discs (10 mm in diameter) with the leaf puncher and place the discs on water-soaked cotton in the cup.
4.3.2.9. Transfer the recovered adults onto the leaf discs (1 adult/disc) with the brush.
4.3.2.10. After drying, cover the cup with the vented lid and incubate at 16:8 L:D and 26°C.

## Results

### Synchronization of egg hatching/larval emergence

**Fig 3:**
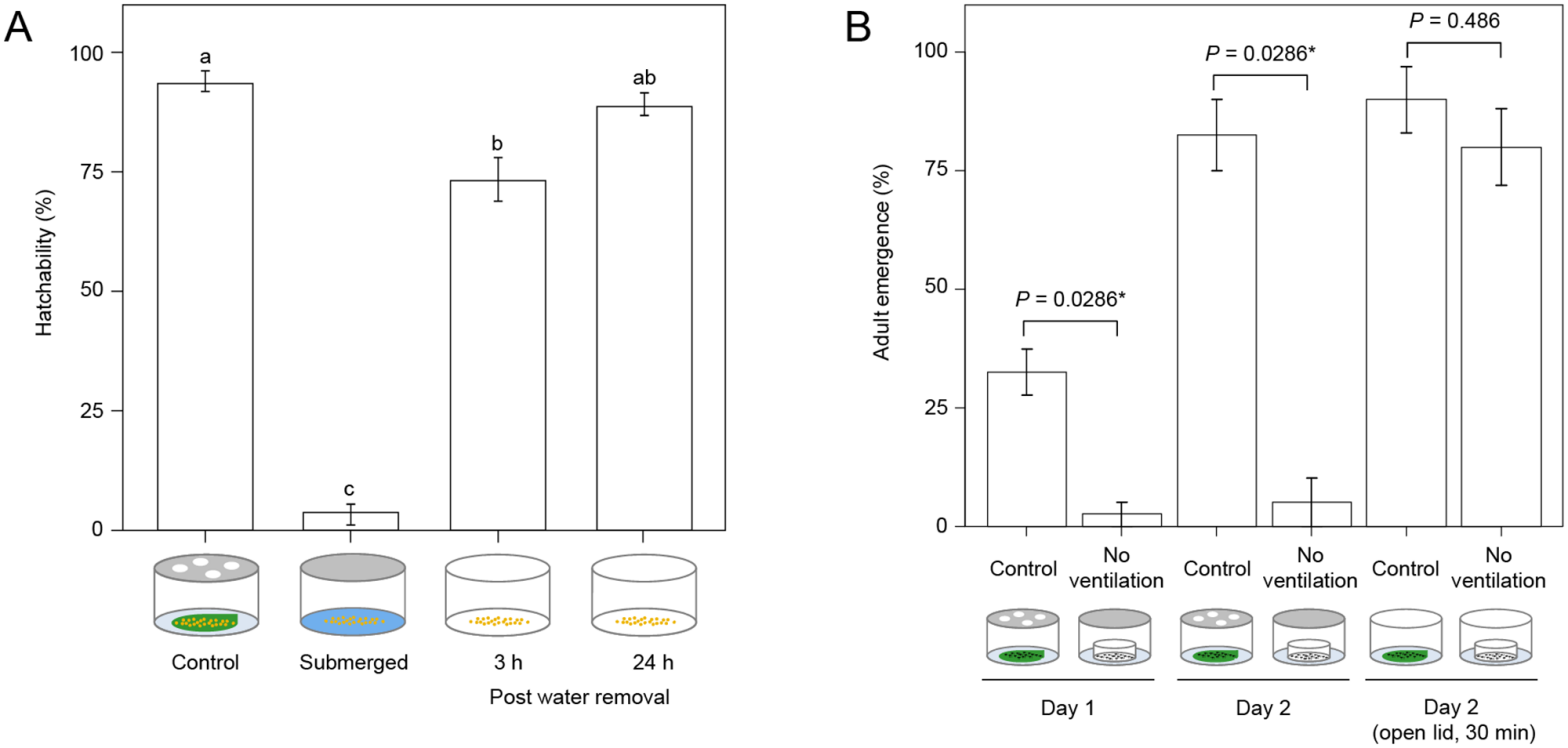
Synchronization of mite development. (A) Synchronization of egg hatching. Relative egg hatchability in: Control, 4-day-old eggs in 50% RH (*n* = 120–271 with 3 replicates); Submerged, eggs submerged in water for 4 days (*n* = 86–142 with 3 replicates); Post water removal, eggs submerged in water for 4 days, then drained and incubated for 3 and 24 h in 50% RH (*n* = 85–137 with 3 replicates). All samples were incubated at 26°C. Data are shown as mean ± SE. Values with different letters differ significantly in the cumulative hatchability (*p*<0.05, Tukey’s HSD test after one-way ANOVA). (B) Relative adult emergence from deutonymphs under different environments (*n* = 10 with 4 replicates). Day 1 and Day 2, adult emergence from deutonymphs kept at 18°C in cups that were covered with vented (Control) and non-vented (No ventilation) lids after 1 and 2 days respectively. Day 2 (open lid, 30 min), adult emergence from deutonymphs kept at 18°C in cups that were covered with vented (Control) and non-vented (No ventilation) lids for 2 days and transferred to 26°C and 50% relative humidity environment for 30 min. Data are shown as mean ± SE. Data at each time point were compared using the Wilcoxon–Mann–Whitney test (no asterisk, *p* > 0.05; *, *p* < 0.05).

The efficiency of synchronization of egg hatching protocol was evaluated by comparing hatchability of eggs that were: a) deposited on bean leaf, b) submerged in water for 4 days, and c) initially submerged in water for 4 days and were subsequently moved to 50% RH for 3 and 24 h, Fig 3A. In the control treatment, adult female mites deposited eggs on a bean leaf. After 1 day, female mites were removed and eggs were incubated at 50% RH. After 3 days, 93.9 ± 2.0% (mean ± SE) of eggs in the control treatment hatched (*n* = 120–271 with 3 replicates). In contrast, only 3.0 ± 2.0% of eggs of the same age that were submerged in water hatched (*n* = 86–142 with 3 replicates). However, upon water removal, 73.2 ± 4.6% and 89.3 ± 2.2% of these eggs hatched in first 3 and 24 h, respectively (*n* = 85–137 with 3 replicates). The majority of larvae emerged within the first 3 h after water removal, yielding a larval population that is tightly synchronized. The hatchability within the first 24 h after water removal was comparable to that in the control (*p*>0.05). S1 Video shows the initial 90 minutes of egg hatching upon draining the water from eggs that were submerged for 4 days.

### Synchronization of adult emergence

Small differences in RH dramatically impact mite molting. In the Control treatment (cups with vented lid), the RH was 96%, while in No ventilation treatment (in cups with non-vented lids) the RH was 100%. RH was measured with a sensor (HHA-3151, T&D, Nagano, Japan) placed inside the cup. In the Control treatment, 33% and 83% adults emerged 1 and 2 days after deutonymphs became quiescent, respectively. These ratios dropped to 3% and 5% in the No ventilation samples (Fig 3B). When the cups containing the same 2-day teleiochrysalis were moved for 30 minutes, without lids, in a chamber with 50% RH, 90% and 80% of the adults have emerged in the Control and No ventilation treatments, respectively (Fig 3B). Almost all teleiochrysalis females in No ventilation samples molted within 3 h, yielding a uniform population of freshly emerged adults. Thus, ambient relative humidity is a prominent factor controlling the molting of deutonymph and is useful in generating synchronized adult cohorts. S2 Video shows adult molting in No ventilation samples within the first 30 minutes after incubating deutonymphs into 50% RH environment.

### Methods for delivery of small molecules

#### Artificial diet

**Fig 4:**
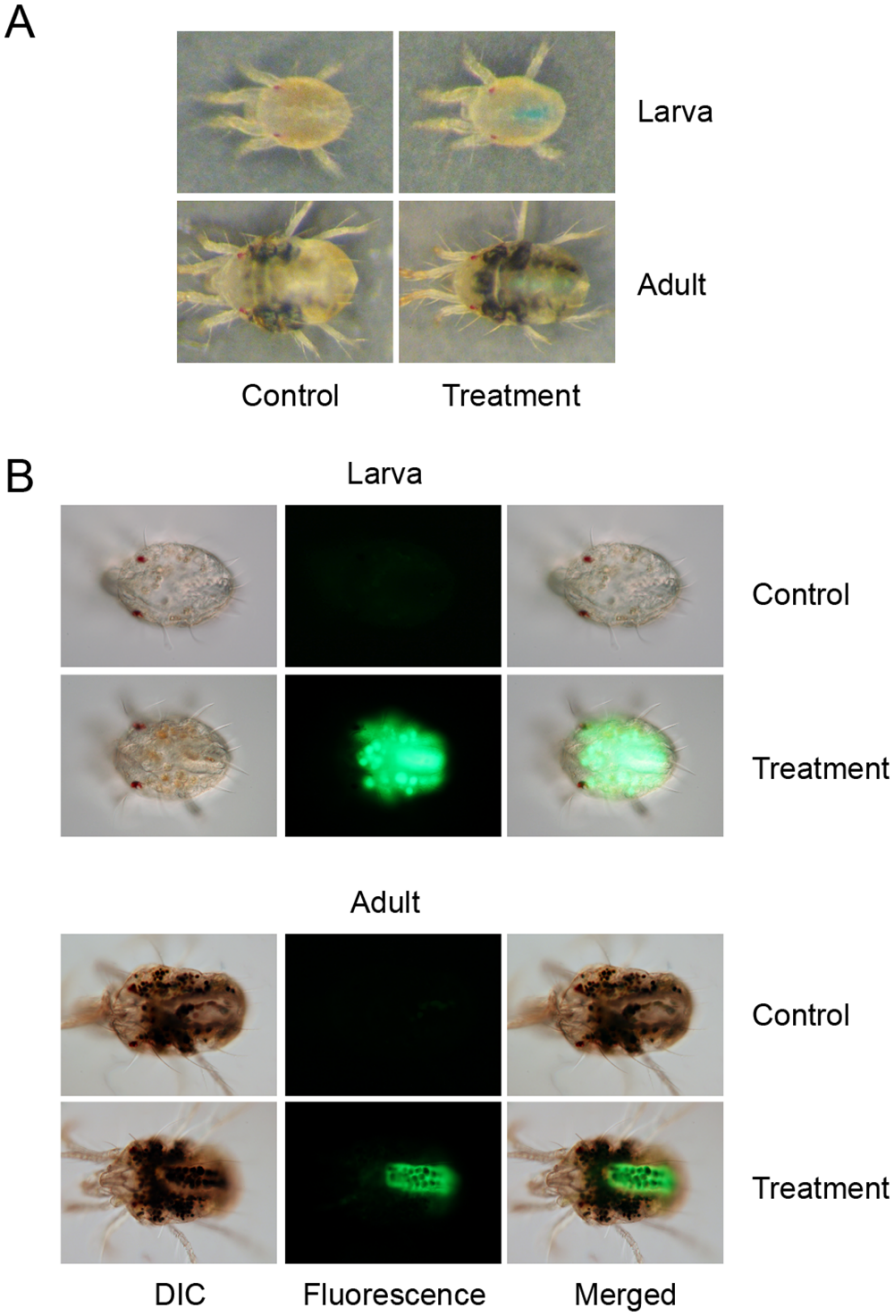
Spider mite larvae and adult females feeding for 24 h on an artificial diet delivered through Parafilm^®^ M hemispheres. (A) Diet supplemented with the 3% blue food dye. (B) Diet supplemented with 100 ng/μL Alexa Fluor 488 green-fluorescent dye. Mite images in (A) represent the control on the left and mites feeding in the presence of the 3% blue food dye on the right. Images in (B) were taken with an exposure time of 0.4 sec (ISO: 400). Differential interference contrast (DIC) and fluorescent images were merged at 50% opacity with ImageJ in (B) [32].

**Fig 5:**
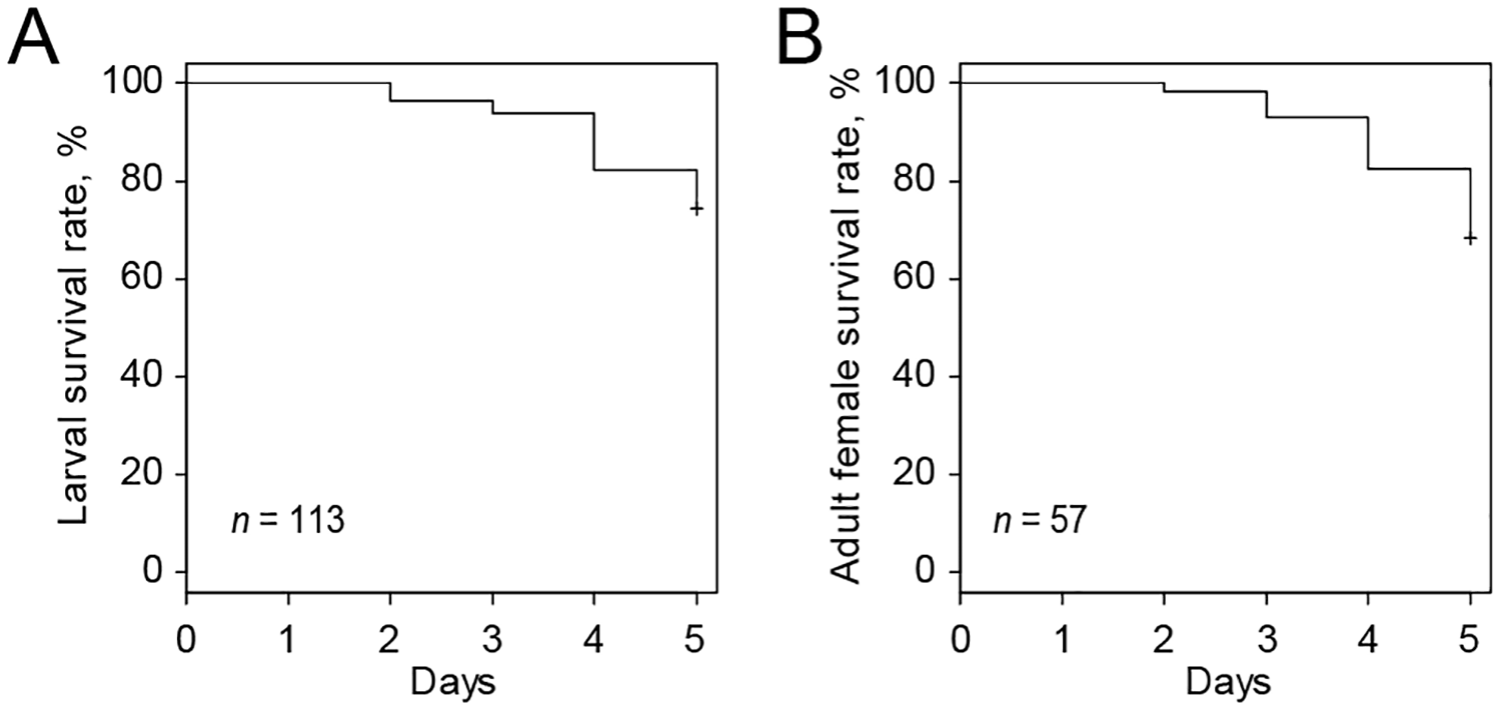
Analysis of the mite survivorship during a 5-day feeding period on artificial diet. (A) Survival of newly-hatched larvae (*n* = 113). (B) Survival of adult females (*n* = 57). Survival curves were plotted with the Kaplan–Meier method.

**Note**: Mite containment was not perfect and the rate of mite escapes from the diet-filled hemispheres partially reduced the mite population size. For example, 22% escape rate was observed for larvae (*n* = 65). Adults were better retained with only 6% of individuals (*n* = 65) escaping after 5 days of feeding on hemispheres surrounded by a water barrier.

Several parameters of mite development and physiology, while feeding on the artificial diet, were determined:

1. **Ability of the artificial diet to support spider mite development** throughout its life cycle was tested by placing 50 females on a cluster of 4 hemispheres. After 24 h, females were removed and around 100 eggs were kept on diet-filled Parafilm^®^ M hemispheres at 16:8 L:D, 26°C and 50% RH. Hemispheres filled with the fresh diet were replaced every 4 days. Mites progressed through all developmental stages (protonymph, deutonymph and adult) and reached adulthood. Mite development occurred at a slower rate relative to bean-leaf-reared larvae, as adults emerged at day 12 instead of day 9, respectively.
  Emerged females were not able to lay eggs. However, when placed on bean leaves they regained fecundity. Males reared on the artificial diet had normal mating behavior (see S3 Video) and fertilized newly-emerged females reared on bean leaves that yielded progeny with a normal sex ratio (f:m) of 7:3 (*n* = 96).
2. **Ingestion of the artificial diet** and diet distribution within the spider mite body was confirmed in mites reared on beans that were subsequently transferred to feeding on an artificial diet for 24 h (Fig 4). For this purpose, the diet was supplemented with 2 different tracers: a 3% blue food dye or 100 ng/μL of the Alexa Fluor 488 fluorescent dye. Dyes were added to the diet solution immediately before making of diet hemispheres. Mites fed with the fluorescent dye were washed with 0.1% Triton X-100 post-feeding and mounted in a solution of 50% glycerol in PBS buffer (v/v) for observation under an epifluorescence microscope (Zeiss, Oberkochen, Germany). As shown in Fig 4, both dyes localize within the mite digestive tract.
3. **Larval and adult female survivorship** while feeding on an artificial diet for 5 days was assessed by following the mortality of larvae and adults. Both developmental stages had similar survivorship of ~80% in a 5-day period (Fig 5).

#### Leaf coating method

This method is designed to evenly apply a defined amount of experimental solution on leaf discs of known surface area. It mimics the delivery of compounds by spraying but does not require specialized equipment such as a Potter spray tower. As two-spotted spider mite feeds from the cell content of the individual mesophyll cells that are internal to the leaf tissue [33], the presence of a surfactant facilitates the spread of the experimental solution on the leaf and its penetration into the leaf tissue. We tested Silwet L-77, a surfactant shown to be a critical component of *Agrobacterium* suspension solutions for plant transformation that enhances solution infiltration into the plant tissues [34]. According to the recommended concentration range, we tested the potential toxic or deterring effects of Silwet L-77 on mites. Bean leaf discs (10 mm in diameter) were coated with 6 μL of water or 0.0125%, 0.025% and 0.05% of Silwet L-77. Individual newly-molted female adult mites were placed on the bean leaf discs to measure mite mortality and escape percentages at 24 and 48 h of exposure. Non-treated leaf discs were used as the control. We also noted the spreading of the aqueous solution on the leaf surface and the timing of solution absorbance by the leaf tissue as a function of Silwet L-77 concentration. As shown in Table 1, <3% (*n* = 22–25 with 3 replicates) of adults escaped from leaf discs coated with water or 0.0125–0.025% of Silwet L-77 and the percentages were comparable to the control (0%; *n* = 25 with 3 replicates) at 48 h of exposure (*p*>0.05). A slight higher percentage (9.3±5.8%; *n* = 23–25 with 3 replicates) of adults escaped from leaf discs coated with 0.05% of Silwet L-77 but it was also comparable to the control at 48 h of exposure (*p*>0.05). After 48 h of exposure, no mite mortality was recorded on leaf discs coated with Silwet L-77 at 0.025% (data not shown) that also showed the best solution spreading and mite retention rate (Table 1). Thus, we used 0.025% Silwet L-77 in the protocol.

**Table 1:** Effect of Silwet L-77 on solution properties and mite behavior at 24 and 48 h after feeding on leaf discs coated with water or Silwet L-77 (0.0125, 0.025 and 0.05%, v/v) at 26°C.

Treatments	Spread time (min)	Absorption/drying time (min)	% Escape	
			24 h	48 h
No treatment	–	–	0.0	0.0
Water	NA	NA	2.7 ± 2.7	2.7 ± 2.7
Silwet L-77, 0.0125%	3–4	9–11	0.0	0.0
Silwet L-77, 0.025%	~1	6–8	2.7 ± 1.3	2.7 ± 1.3
Silwet L-77, 0.05%	<1	6–7	9.3 ± 5.8	9.3 ± 5.8

Data for the escape percentage were collected from 3 replicates (*n* = 22–25 females per replicate) and were arcsine square-root transformed before the statistical analysis. All data are shown as mean ± SE (except data for which SE was zero). No significant differences among treatments were detected in each column with *p* > 0.05 (Tukey’s HSD test after one-way ANOVA).

Developed leaf coating protocol was tested for its ability to deliver small molecules into the mites and to support mite larval and adult development.

**Fig 6:**
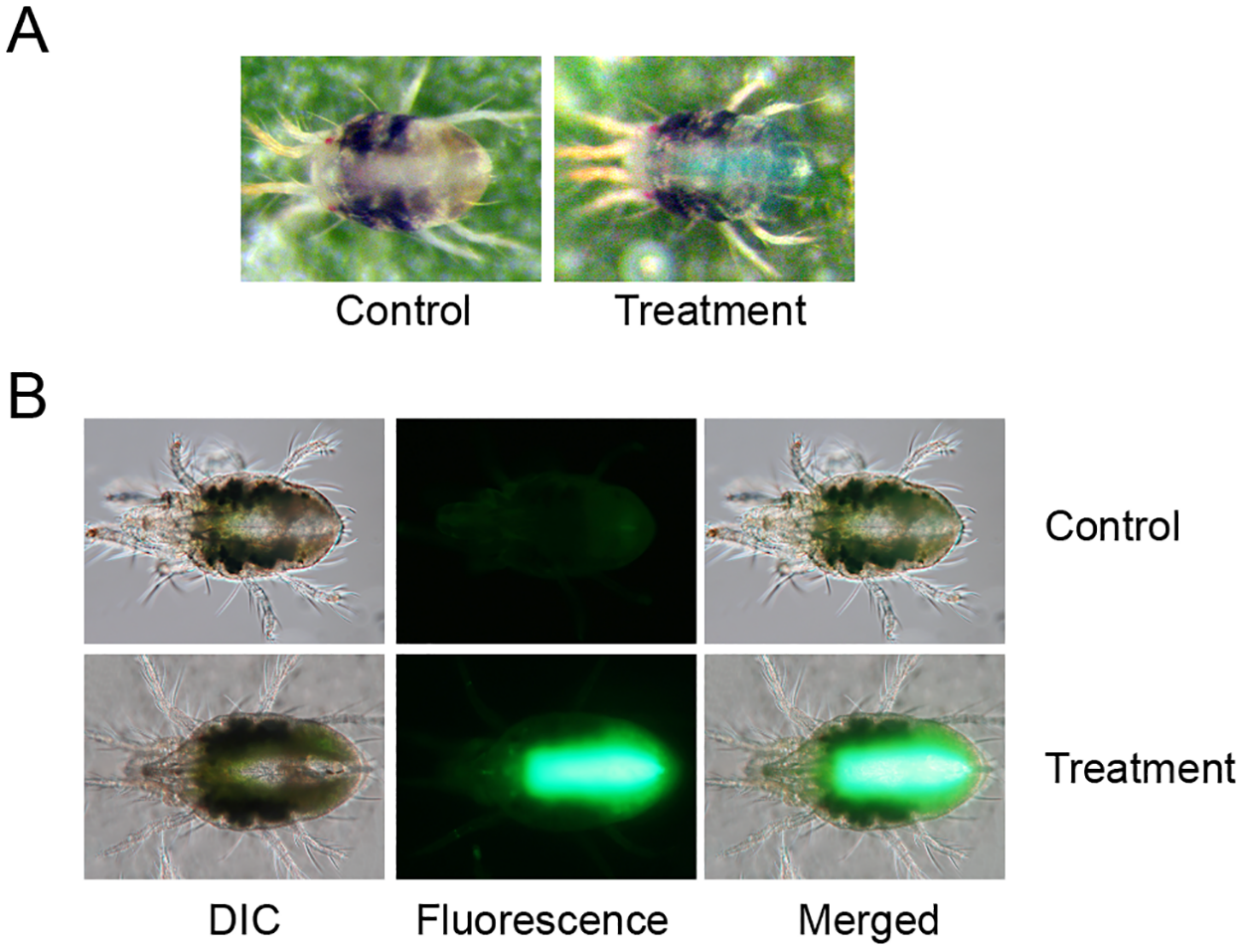
Accumulation of tracer dyes in adult female mites after feeding for 48 h on a leaf disc. Leaf disc was coated with: (A) water (left, Control) and 3% blue food dye (right, Treatment); or, (B) fluorescent Alexa Fluor 488 dye (100 ng/μL). Images in (B) were taken with an exposure time of 0.4 sec (ISO: 400). Differential interference contrast (DIC) and fluorescent images were merged at 50% opacity with ImageJ [32].

**Fig 7:**
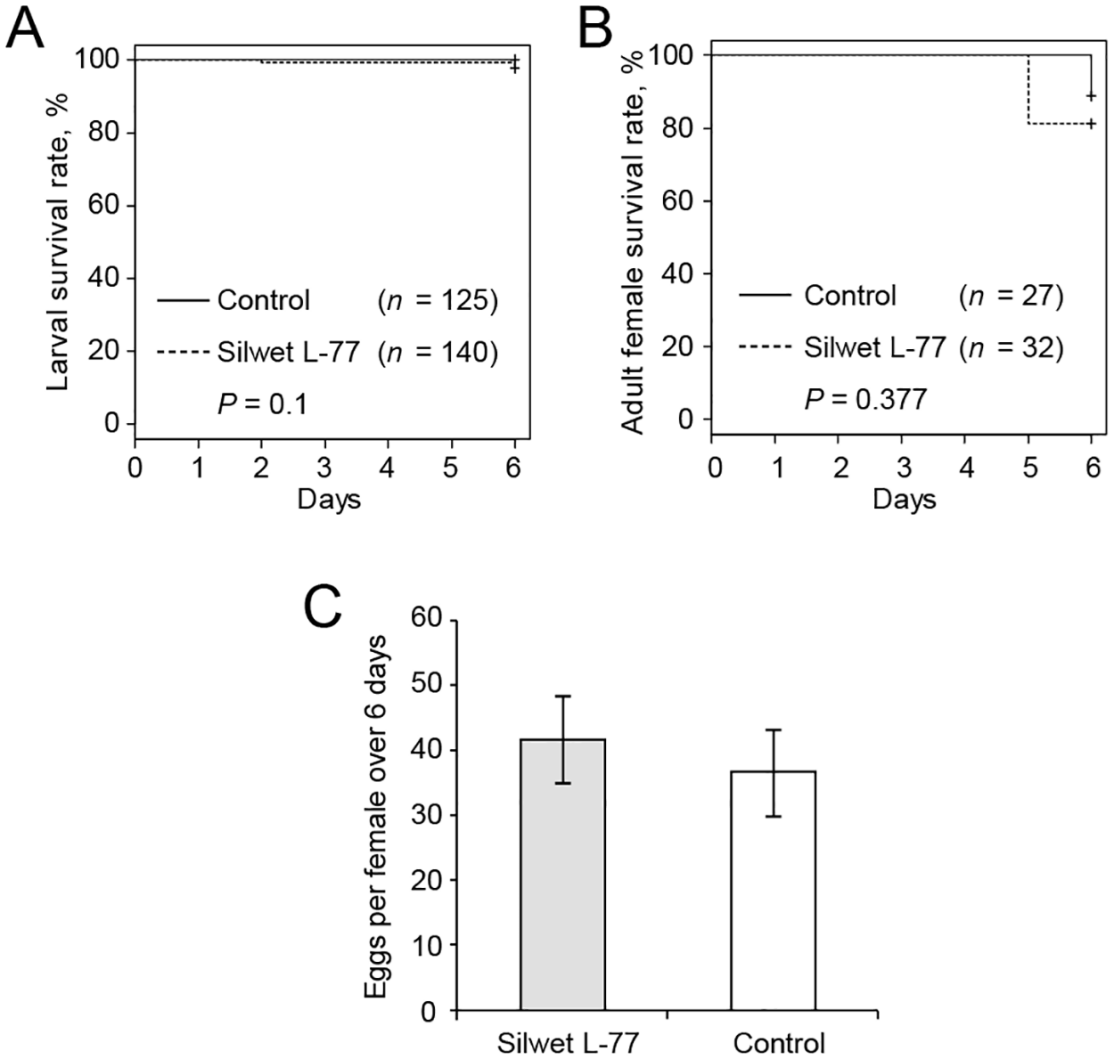
Analysis of mite performance while feeding on bean leaf discs coated with water or Silwet L-77. Larval (A) and adult female (B) survivorship during a 6-day feeding on bean leaf discs coated with water or Silwet L-77. Sample: (A) water control (solid line, *n* = 125; 0.025% Silwet L-77 (dashed line, *n* = 140); (B) water control (black line, *n* = 27); 0.025% Silwet L-77 (dashed line, *n* = 32). Survival curves were plotted with the Kaplan–Meier method and compared with the log-rank test. (C) Fecundity of females during a 6-days feeding period on bean leaf discs coated with water (*n* = 10) or 0.025% Silwet L-77 (*n* = 15). Results are shown as mean ± SE. Difference between treatments was tested with *t*-test, *P* = 1. Coated leaf discs were replaced every other day (A-C).

1. **Ingestion of the solution** applied on the leaf surface was confirmed with solutions supplemented either with 3% of the blue food dye or 100 ng/μL Alexa Fluor 488 fluorescent dye. The localization of tracer dyes in mite body was determined 48 h after feeding started. For each treatment, 30 freshly-molted females (prepared as described in Protocol 3) were transferred to 6 leaf discs coated with tracer dyes or water. Mites that fed on bean leaf discs coated with the florescent dye were washed in 0.1% Triton X-100 and mounted in a solution of 50% glycerol in PBS buffer (v/v) for observation under an epifluorescence microscope (Zeiss, Oberkochen, Germany). Both tracer dyes were detected in the mite gut (Fig 6) indicating that the application of the solution on leaf surface does deliver experimental compounds into the mite digestive system. While all mites displayed fluorescent staining in their gut (that is a sensitive detection of a tracer intake), only 60% of mites were blue. This indicates the variability in the tracer dye intake that should be taken into account when delivering small molecules.
2. **Mite performance while feeding on coated leaf discs—including larval and adult female survivorships and fecundity (5 larvae/disc and 1 adult/disc)**—were determined over 6 days to investigate whether Silwet L-77 may have any long-term effects. As shown in Fig 7, inclusion of 0.025% Silwet L-77 in the experimental solution did not affect mite viability nor female fecundity.

#### Soaking

Soaking is an established method for delivery of small molecules (e.g. dsRNA) to *Caenorhabditis elegans* Maupas (Rhabditidae) or insect cell lines [35–38], but has been exploited in insects and other arthropods in only few cases [17,39,40]. Thus, the main objective in the development of soaking as a delivery method for spider mites was to secure mite survivorship upon submergence and to ensure penetration of the solution into the mite body. As aqueous solutions have high surface tension preventing mite submergence, we tested the survival of newly-hatched larvae and newly-molted adults in aqueous solutions that contained either 0.1% Tween 20 or 0.1% Triton X-100. We focused on a narrow interval of mite development when the chitin exoskeleton is resynthesized with the aim to increase the penetrability of the solution into the body of the mite.

As shown in Table 2, 77.2±7.2% and 70.4±8.0% (*n* = 19–25 with 5 replicates) or 47.9±3.5% and 45.4±3.4% (*n* = 20–25 with 5 replicates) of newly-molted adult female mites survived at 24 and 72 h after recovery from soaking at 20°C for 24 h in 0.1% Tween 20 or 0.1% Triton X-100, respectively. As shown in Table 3, 71.9±2.9% and 65.0±5.2% (*n* = 23–30 with 5 replicates) or 62.1±4.4% and 51.3±5.7% (*n* = 15–36 with 5 replicates) of newly-hatched larvae survived at 24 and 72 h after recovery from soaking at 20°C for 24 h in 0.1% Tween 20 or 0.1% Triton X-100, respectively. Shortening the soaking time to 4 h increased the larval survivorships to 86.1±3.1% and 77.7±3.5% (*n* = 25–30 with 5 replicates) or 78.0±4.2% and 68.4±5.4% (*n* = 25–29 with 5 replicates) in 0.1% Tween 20 or 0.1% Triton X-100, respectively. Further shortening the soaking time to 2 h increased the larval survivorships to 88.9±2.8% and 86.6±3.4% (*n* = 23–28 with 5 replicates) or 76.8±4.8% and 63.2±7.5% (*n* = 25–32 with 5 replicates) in 0.1% Tween 20 or 0.1% Triton X-100, respectively. The survivorship of larvae at 72 h after recovery from 2 h soaking in 0.1% Tween 20 was comparable to that (95.5±0.7%, *n* = 29–32 with 5 replicates) in control (*p*>0.05). These data establish the use of Tween 20 and the duration of about 2–4 h as appropriate parameters to achieve >70% survivorship for soaking larvae, while soaking adults can be extended to 24 h.

**Table 2:** Survival of adult *T*. *urticae* females at 24 and 72 h after recovery from soaking in 0.1% Tween 20 or 0.1% Triton X-100 (v/v) at 20°C for 24 h.

Soaking duration (h)	Surfactant	% Survival	
		24 h	72 h
0	–	100 a	99.2 ± 0.8 a
24	0.1% Tween 20	77.2 ± 7.2 b	70.4 ± 8.0 b
24	0.1% Triton X-100	47.9 ± 3.5 c	45.4 ± 3.4 c

Data for the survival were collected from 5 replicates (*n* = 19–25 females per replicate) and were arcsine square-root transformed before the statistical analysis. All data are shown as mean ± SE (except data for which SE was zero). Different letters within a column show a significant difference with *p* < 0.05 (Tukey’s HSD test after one-way ANOVA).

**Table 3:** Survival of *T*. *urticae* larvae at 24 and 72 h after recovery from soaking in 0.1% Tween 20 or 0.1% Triton X-100 (v/v) at 20°C for 0, 2, 4, or 24 h.

Surfactant	Soaking duration (h)	% Survival	
		24 h	72 h
–	0	98.1 ± 0.8 a	95.5 ± 0.7 a
0.1% Tween 20	2	88.9 ± 2.8 b	86.6 ± 3.4 ab
0.1% Tween 20	4	86.1 ± 3.1 bc	77.7 ± 3.5 bc
0.1% Tween 20	24	71.9 ± 2.9 cd	65.0 ± 5.2 cd
0.1% Triton X-100	2	76.8 ± 4.8 bcd	63.2 ± 7.5 cd
0.1% Triton X-100	4	78.0 ± 4.2 bcd	68.4 ± 5.4 cd
0.1% Triton X-100	24	62.1 ± 4.4 d	51.3 ± 5.7 d

Data for the survival were collected from 5 replicates (*n* = 15–36 larvae per replicate) and were arcsine square-root transformed before the statistical analysis. All data are shown as mean ± SE. Different letters within a column show a significant difference with *p* < 0.05 (Tukey’s HSD test after one-way ANOVA).

**Fig 8:**
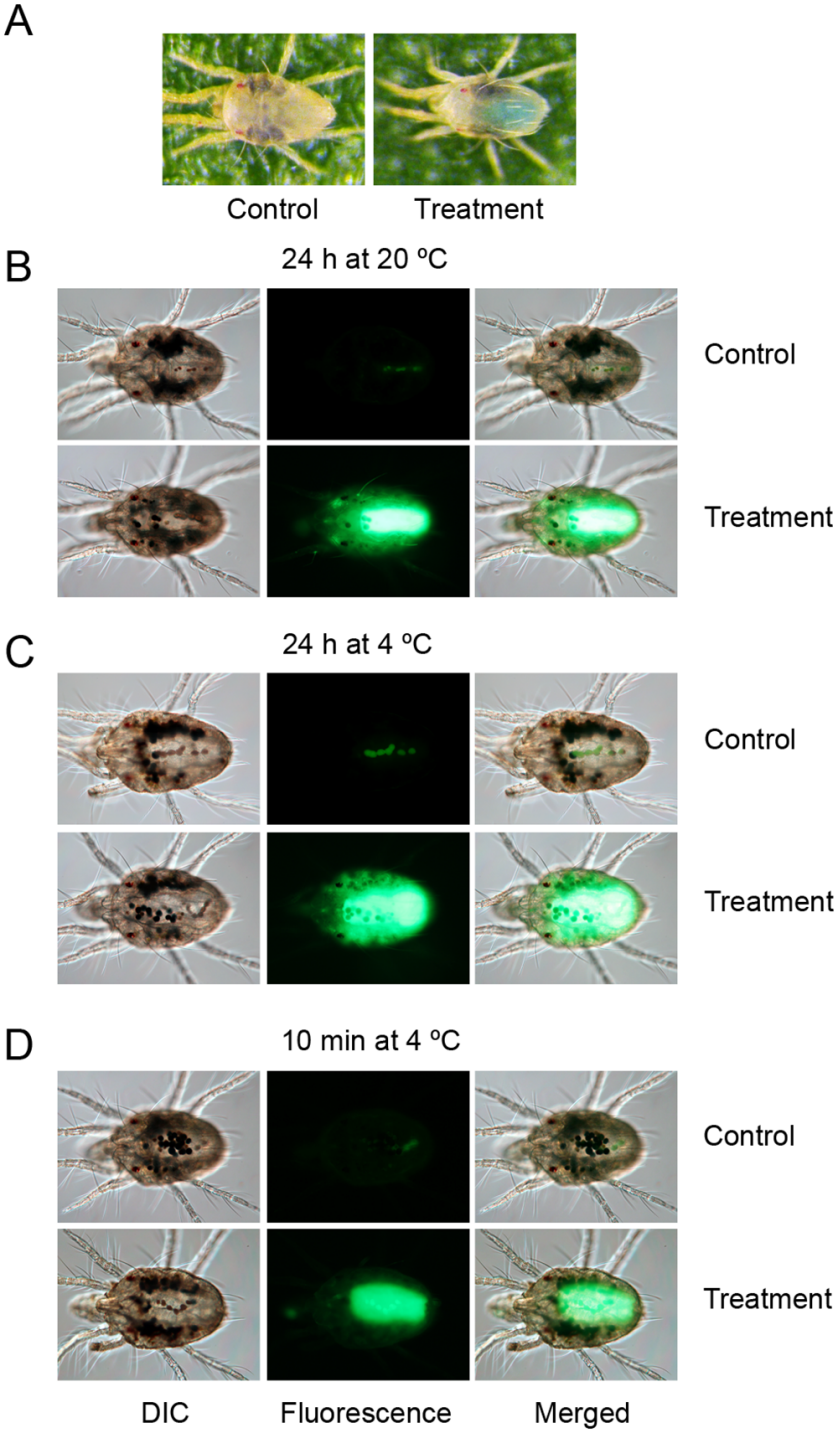
Accumulation of tracer dyes in mites after recovery from soaking. (A) for 24 h at 20°C, in 0.1% Triton X-100 (Control) and blue food dye (0.1% Triton X-100 + 3% blue food dye); (B) for 24 h at 20°C, in 0.1% Triton X-100 (Control) and fluorescent dye (0.1% Triton X-100 + 100 ng/μL Alexa Fluor 488); (C) for 24 h at 4°C, in 0.1% Triton X-100 (Control) and fluorescent dye (0.1% Triton X-100 + 100 ng/μL Alexa Fluor 488); and (D) for 10 minutes at 4°C, in 0.1% Triton X-100 (Control) and fluorescent dye (0.1% Triton X-100 + 100 ng/μL Alexa Fluor 488). The fluorescent images (in B-D) were taken with and an exposure time of 0.4 s (ISO: 400). The differential interference contrast (DIC) and fluorescent images were merged at opacity of 50% with ImageJ [32].

To test the efficiency of the soaking method to deliver small molecules, we used blue food dye and the Alexa Fluor 488 fluorescent dye as tracers to monitor the uptake of compounds by larvae and adults. We also tested the effect of soaking duration (24 h and 10 minutes) and temperature (soaking at 20°C and 4°C) on the uptake of the fluorescent dye by adult mites. Mites soaked in the solution containing the fluorescence dye were washed after soaking with 0.1% Triton X-100, were mounted in 50% glycerol in PBS on a slide and were observed by an epifluorescence microscope. Tracer dyes could be detected in both larvae (not shown) and newly-molted adults. In adults, the staining can be observed irrespective of the timing and the temperature of soaking (Fig 8). However, brighter fluorescence was observed in mites soaked for 24 h. Tracer dyes could be detected in the mite gut, but the staining appears to extend beyond the alimentary track. Like with the coating method, variability in number of mites stained with tracer dyes could be observed. While majority of mites were stained with the fluorescent dye, 88% of soaked adults (*n* = 32) displayed the blue color, indicating variability in dye intake within the treated mite population.

## Discussion

This work establishes a detailed and standardized set of protocols for delivery of small molecules (e.g. chemicals, dsRNA, tracer dyes) into spider mites, as supplements to an artificial diet, or constituents of a solution that is either applied on a leaf surface (leaf coating) or used for soaking the mites. In addition, we provide a detailed protocol for the preparation of spider mite populations that are tightly synchronized in their development, which is necessary to obtain reproducible results in bioassays.

### Artificial diets for spider mites

Artificial diets adequate to sustain TSSM have been published over 30 years ago [18–20,41] but have not been widely implemented. Most liquid diets formulated for mites and aphids [26,27,29] could keep mites alive, but did not facilitate progression through a full life cycle. Of the diets tested by us, only the diet described by Van Der Geest *et al*. [18] could support a complete developmental cycle of TSSM. The development of mites feeding on this diet was identical in progression but delayed in time relative to mites maintained on bean leaves. Thus, the diet described herein successfully replaces leaf tissues for the oral delivery of small molecules (Fig 6). Although the mortality and dispersal of mites fed on the artificial diet was relatively high when considering the whole life cycle, the diet supported both larvae and adult mites with moderate mortality (~20%) over a 5 day-period (Fig 7).

Female mites reared on this artificial diet were not fecund. However, their ability to lay eggs was restored when transferred to bean leaves, suggesting that the diet supports normal female development but lacks component(s) or signal(s) necessary for oogenesis. Tracing the bean leaf compound(s) that induces/enables mite fecundity would provide important insights into mite oogenesis and would improve the artificial diet. In addition, the identification of compound(s) and processes that are specifically required for mite female oogenesis could be exploited as targets for chemical inhibition, leading to mite contraception as an alternative way to suppress mite population. Despite the failure to support female oogenesis, the same diet supported the normal development of males that were able to mate, produced viable sperm, and gave rise to properly developing diploid female progeny.

Artificial diets have been widely used to deliver dsRNA to insects [8,9]. The diet presented here, based on diet described in [18], was previously applied through the membrane feeding system to deliver dsRNA into *T*. *urticae* [7]. However, the high mortality (>50% over the 3-day experimental period) and contamination of diet by fungi and bacteria prevented its use. We tested the membrane feeding system as well, but found that uneven stretching of Parafilm^®^ M is introducing variability into the experimental system. Thus, the artificial diet protocol includes the pretreatment of Parafilm^®^ M with UV and uses the custom built vacuum device (S1D Fig) to form Parafilm^®^ M hemispheres that are compatible with mite feeding. In addition, protocol incorporates diet sterilization and its replacement every 4 days that is sufficient to prevent microbial contamination.

Besides RNAi studies, artificial diets supplemented with purified plant-derived compounds have been used in the identification of plant defense metabolites, for example to show that indole glucosinolate breakdown products act as feeding inhibitors in the aphid *Myzus persicae* [42,43]. Although other artificial diets tested in this study do not support certain mite developmental transitions, they are still useful for experimental purposes. For example, a chemically defined diet was recently used to collect mite salivary secretions to profile peptides that are secreted into plant tissues during feeding [28].

### Leaf coating as a proxy to natural feeding

Leaf coating is an alternative method for oral delivery in which mites feed in their natural settings. It is thus most suitable for studies involving the fragile larvae. Since mites feed on mesophyll cells located in inner leaf tissues [33], it was important to confirm their ability to ingest tracers applied on the leaf surface. This was indeed demonstrated because the distribution patterns of dyes within the mite body were similar whether ingested through the artificial diet or after application on the leaf surface (compare Figs 6 and 8).

Furthermore, surfactants such as Silwet L-77 practically improve leaf coating methods. They enhance the penetration of solutions into the leaf internal tissues and thus increase the fraction of compounds delivered into mites. Silwet L-77 also promotes the passive and uniform spread of solutions across the leaf disc surface, which is key to streamline protocols for compound screening.

### Soaking, a simple method for spider mite bioassays

Finally, a soaking method has been validated as an alternative protocol for high throughput delivery of small molecules in mites. First, we discovered that mites are resilient to water exposure as eggs and deutonymphs arrest their hatching and molting, respectively, upon submergence. These behaviors were used to prepare tightly synchronized experimental populations of larvae and adults (Protocols 3.1 and 3.2) [24,25]. After optimization, the soaking of larvae and adults in aqueous solution resulted in high survivorship (>70%). In summary, adults sustain soaking for at least 24 h with low mortality. However, larvae only sustain shorter submergence (up to 4 h). Furthermore, larvae and adults were dramatically sensitive to Triton X-100, but only marginally affected by Tween 20.

While soaking is not an oral delivery method *per se*, tracer dyes were detected in the gut of soaked mites, in a pattern similar of those observed when dyes were administered through artificial diet or leaf coating. However, the tracer distribution appears broader suggesting that compounds may be delivered to a wider domain. This hypothesis can be tested experimentally with compounds that affect mite processes in well-defined organs or tissues. Finally, the soaking procedure is inexpensive, rapid, and can be easily scaled-up.

### Perspectives

The 3 methods for which we provide detailed protocols are useful to deliver small molecules into the mite gut, for example chemicals or nucleic acids. They can be adapted to a wide range of applications, including the identification of plant defense compounds specifically targeting TSSM and functional screens based on the perturbation of gene expression initiated in the gut epithelial cells. All methods described are quick and inexpensive, and designed for mid- to high-throughput compound screens that will strengthen and broaden experimental approaches towards a better understanding of TSSM biology.

## Supporting information

S1 Fig Supplies and custom devices used. (A) Modified aquarium air pump with inverted air flow (Whisper 10–30; Tetra, Blacksburg, VA) with 1 mL polypropylene pipette tip for spider mite collection. (B) Polystyrene cup and lid (V-9, As-one, Osaka, Japan). (C) Polystyrene cup with vented lid made using gas-permeable filters with 0.45 micron pore size (Milliseal, EMD Millipore, Billerica, MA). (D) Vacuum device, consisting of a 96-hole well plate (plate thickness 4.2 mm, hole diameter 4.5 mm) fitted on a vacuum manifold plate (Analytical Research Systems, Florida, USA) for artificial diet encapsulation. (E) Individual artificial diet hemispheres in 24-well plate. Food die was added to diet samples. (F) Setup used for larval soaking experiments.
(TIF)

S1 Table Composition of chemicals required for the preparation of 100 mL of the artificial diet. (DOCX)

S1 Video Hatching of *T*. *urticae* eggs. Hatching of *T*. *urticae* eggs at 26°C and 50% RH after being submerged in water for 4 days at 26°C. The recording was conducted over 1.5 h (accelerated 120X) after water removal.
(MP4)

S2 Video Molting of teleiochrysalis females. Adult emergence of *T*. *urticae* induced by decreasing RH from 100% to 50% and increasing air temperature from 18°C to 26°C. The recording was conducted over 30 min (accelerated 120X) after the environmental change. The teleiochrysalis females are molting on a bean leaf in this video, whereas they also molt in an empty Petri dish as in the presented protocol in the text, to avoid unwanted feeding in newly-molted adult females.
(MP4)

S3 Video Mites raised using artificial diet. Newly molted male adult grown on artificial diet mating with a newly emerged bean-reared female.
(MP4)
