# Correction: Protocols for the delivery of small molecules to the two-spotted spider mite, *Tetranychus urticae*

**DOI:** 10.1371/journal.pone.0190025

**Published:** 2017-12-15

**Authors:** Takeshi Suzuki, María Urizarna España, Maria Andreia Nunes, Vladimir Zhurov, Wannes Dermauw, Masahiro Osakabe, Thomas Van Leeuwen, Miodrag Grbic, Vojislava Grbic

The third author, Maria Andreia Nunes, should be noted as having also contributed equally to this work.
