# Supplementary figures and images for: Protocols for the delivery of small molecules to the two-spotted spider mite, Tetranychus urticae
Source: PLoS One. 2017 Jul 7;12(7):e0180658. doi: 10.1371/journal.pone.0180658 (PMC5501582; doi:10.1371/journal.pone.0180658)

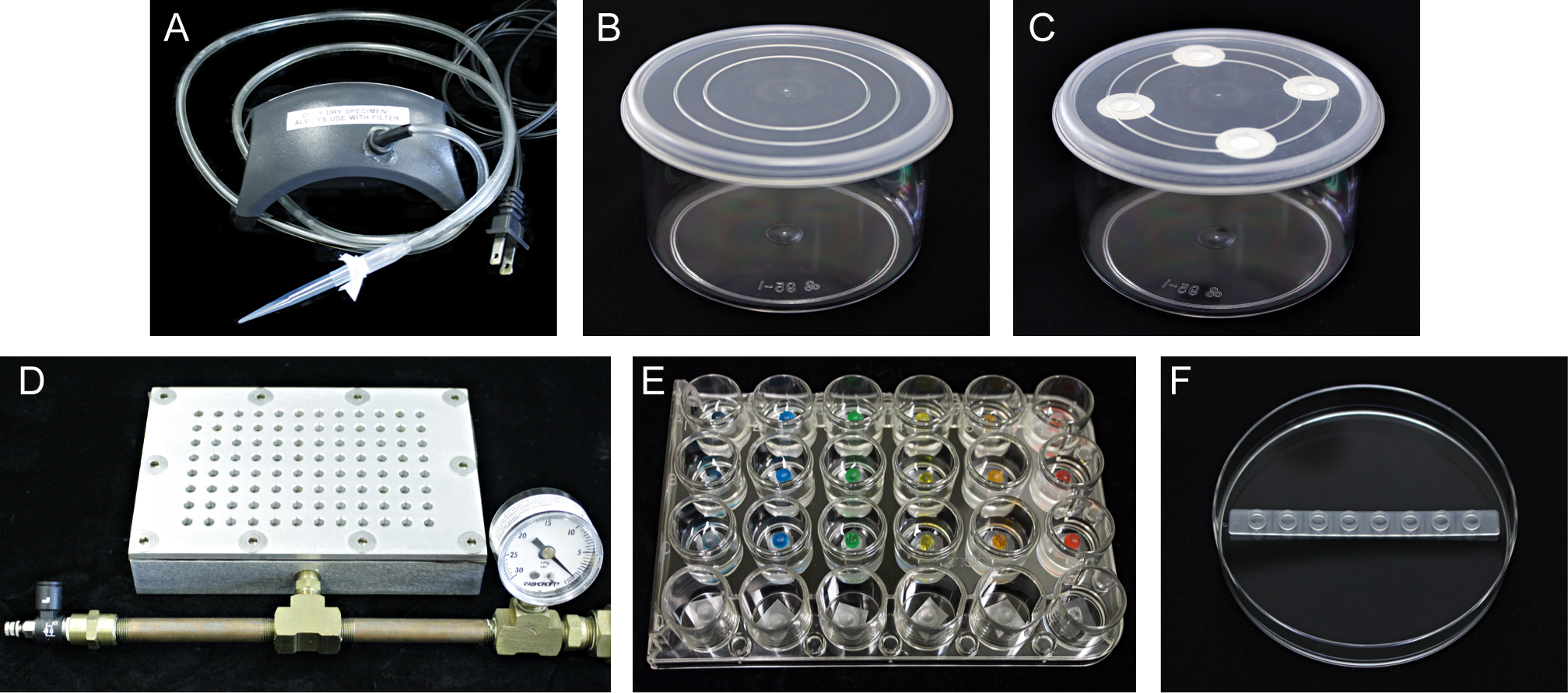

Supplement: S1 Fig — (A) Modified aquarium air pump with inverted air flow (Whisper 10–30; Tetra, Blacksburg, VA) with 1 mL polypropylene pipette tip for spider mite collection. (B) Polystyrene cup and lid (V-9, As-one, Osaka, Japan). (C) Polystyrene cup with vented lid made using gas-permeable filters with 0.45 micron pore size (Milliseal, EMD Millipore, Billerica, MA). (D) Vacuum device, consisting of a 96-hole well plate (plate thickness 4.2 mm, hole diameter 4.5 mm) fitted on a vacuum manifold plate (Analytical Research Systems, Florida, USA) for artificial diet encapsulation. (E) Individual artificial diet hemispheres in 24-well plate. Food die was added to diet samples. (F) Setup used for larval soaking experiments. (TIF) [file pone.0180658.s001.tif]
