# Supplementary material for: Protocols for the delivery of small molecules to the two-spotted spider mite, Tetranychus urticae
Source: PLoS One. 2017 Jul 7;12(7):e0180658. doi: 10.1371/journal.pone.0180658 (PMC5501582; doi:10.1371/journal.pone.0180658)
Supplement: S1 Table — (DOCX) [file pone.0180658.s002.docx]

**S1 Table. Composition of chemicals required for the preparation of 100 mL of the artificial diet.** All catalog numbers are for Sigma-Aldrich (St. Louis, MO) unless otherwise noted.

| **Component** | **Catalog number** | | **Amount** |
| --- | --- | --- | --- |
| **Amino acids** | | | |
| L-Alanine | | A5006 | 4 mg |
| ß-Alanine | | 146064 | 4 mg |
| L-Arginine · HCl | | A5006 | 12 mg |
| L-Asparagine · H2O | | A8381 | 26 mg |
| L-Aspartic acid | | A9256 | 8 mg |
| L-a-amino-n-butyric acid | | D0506 | 2 mg |
| ?-Amino-n-butyric acid | | D8131 | 3 mg |
| DL-Carnithine · HCl | | C9500 | 3 mg |
| L-Citrulline | | C7629 | 3 mg |
| L-Cysteine · HCl | | C1276 | 4 mg |
| L-Cystine | | C7602 | 3 mg |
| L-Glutamic acid | | G1251 | 4 mg |
| L-Glutamine | | G3126 | 12 mg |
| L-Glycine | | 410225 | 3 mg |
| L-Histidine · HCl · H2O | | H8125 | 12 mg |
| L-Homoserine | | H6515 | 3 mg |
| L-Hydroxyproline | | H54409 | 3 mg |
| L-Isoleucine | | I2752 | 12 mg |
| L-Leucine | | L8000 | 8 mg |
| L-Lysine · HCl | | L5626 | 8 mg |
| L-Methionine | | 64319 | 12 mg |
| L-Ornithine · HCl | | O2375 | 8 mg |
| L-Phenylalanine | | 8 mg |  |
| L-Proline | | 81709 | 7 mg |
| L-Serine | | 84959 | 3 mg |
| L-Threonine | | T8625 | 7 mg |
| L-Tryptophane | | T0254 | 4 mg |
| L-Tyrosine | | T3754 | 8 mg |
| L-Valine | | V0500 | 8 mg |
| **Vitamins** | | | |
| Nicotinamide | | 72340 | 0.5 mg |
| (+)-Pantothenic acid, Ca salt | | C8731 | 0.3 mg |
| Aneurine · HCl | | T4625 | 0.1 mg |
| Riboflavin | | R7649 | 0.05 mg |
| Pyridoxine · HCl | | P9755 | 0.3 mg |
| Folic acid | | F7876 | 0.005 mg |
| D-Biotin | | B4501 | 0.005 mg |
| Cyanocobalamin | | C3607 | 0.003 mg |
| para-Aminobenzoic acid | | A9878 | 0.1 mg |
| meso-Inositol | | I5125 | 3 mg |
| Choline chloride | | 26980 | 3 mg |
| L-Ascorbic acid | | A5960 | 50 mg |
| **Mineral salts** | | | |
| K2HPO4 | | 1551128 | 11.4 mg |
| MgSO4 | | M7506 | 4 mg |
| CaCl2 · 2H2O | | C5080 | 2 mg |
| CoCl2 · 6H2O | | C8661 | 0.05 mg |
| CuCl2 · 2H2O | | 307483 | 0.08 mg |
| FeCl3 · 6H2O | | 236489 | 0.6 mg |
| MnCl2 · 4H2O | | M5005 | 0.2 mg |
| ZnCl2 | | 229997 | 0.4 mg |
| **Other components** | | | |
| Cholesterol | | C8767 | 10 mg |
| Casein, Na salt | | C8654 | 2000 mg |
| D-Glucose | | G7021 | 1000 mg |
| Wheat germ powder | | W0125 | 2000 mg |
